# Aorto-Left Ventricular Tunnel: The First Systematic Review of An Uncommon Entity (177 Worldwide Cases from 1965 to 2024)

**DOI:** 10.31083/RCM26005

**Published:** 2025-02-20

**Authors:** Pier Paolo Bassareo, Aurelio Secinaro, Paolo Ciliberti, Marco Alfonso Perrone, Niall Linnane, Sophie Duignan, Paolo Ferrero, Massimo Chessa, Kevin Patrick Walsh, Colin Joseph Mcmahon

**Affiliations:** ^1^Mater Misericordiae University Hospital, National Adult Congenital Heart Disease Service, D07 R2WY Dublin, Ireland; ^2^Children’s Health Ireland at Crumlin, D12 N512 Dublin, Ireland; ^3^School of Medicine, University College of Dublin, D04 V1W8 Dublin, Ireland; ^4^Advanced Cardiovascular Imaging Unit, Bambino Gesù Children’s Hospital, IRCCS, 00146 Rome, Italy; ^5^Department of Cardiac Surgery, Cardiology, Heart and Lung Transplantation Bambino Gesu’ Children’s Hospital, IRCCS, 00146 Rome, Italy; ^6^Division of Cardiology and CardioLab, Department of Clinical Sciences and Translational Medicine, University of Rome Tor Vergata, 00133 Rome, Italy; ^7^Adult Congenital Heart Disease UNIT, Paediatric and Adult Congenital Heart Centre, IRCCS-Policlinico San Donato, San Donato Milanese, 20097 Milan, Italy; ^8^School of Medicine, Vita Salute San Raffaele University, 20132 Milan, Italy

**Keywords:** aorto-to-left ventricular tunnel, echocardiography, cardiac catheterization, computed tomography, magnetic resonance imaging, systematic review

## Abstract

**Background:**

The study was aimed at assessing clinical status and outcome of patients affected by aorto-left ventricular tunnel (ALVT).

**Methods:**

A systematic search of keywords relating to ALVT was conducted to identify papers published between 1965 and February 2024 present on Pubmed/Medline and Scopus.

**Results:**

A total of 109 studies, which in all consisted of case reports and case series comprising 177 patients (64.2% males, *p* < 0.02) met the inclusion criteria. The median age of patients was 9.5 ± 8.9 years. Initial diagnosis was based on echocardiographic findings in 86.4% of patients, and confirmed by computed tomography (CT) and/or magnetic resonance imaging (MRI) in 17%. Of the 177 patients identified, 47.1% were diagnosed with a heart murmur and 32.4% with congestive heart failure. Associated cardiac abnormalities were detected in 39.8% (unicuspid/bicuspid aortic valve with or without stenosis/atresia in 14.8%, coronary artery abnormalities in 9.6%). A total of 90.3% of patients underwent surgery, whilst 4.5% were treated by means of transcatheter closure. Outcomes were largely favorable (death was reported in 5.7%). Mild residual aortic regurgitation continued to be present in 22.7% of the sample. In terms of statistics, no risk factors for death were found.

**Conclusions:**

ALVT, an extremely rare congenital cardiac abnormality, may be diagnosed in both newborns and adults. Initial diagnostic observations are usually made using echocardiography, and subsequently refined by means of catheterization, CT or MRI. Surgery should be performed as soon as possible following diagnosis, particularly due to the inefficacy of medical treatment. In selected cases, transcatheter closure may represent a valid option. The condition is associated with a high mortality rate. Moreover, complications, particularly in the form of residual aortic valve regurgitation, may hamper postoperative prognosis. Due to the rarity of the disease, the setting up of an international registry is recommended.

## 1. Introduction

Aorto-ventricular tunnel (ALVT) presents as an extracardiac channel that passes outside the heart, linking the ascending aorta beyond the sinotubular junction to the left (90%) or right (10%) ventricular chamber [1]. In the vast majority of cases, the aortic opening of tunnels is situated above the right coronary sinus of Valsalva. Accordingly, the tunnel gains access to the left ventricle at the fibrous triangle situated below the inter-coronary commissure, or into the right ventricle either above or below the sub-pulmonary infundibulum. In ALVT, by far the most common form of the disease, the right coronary leaflet of the aortic valve is left devoid of support across part of its hinge-point and thus stems from a strip of fibrous tissue that extends across the aortic root [2]. Tunnels located above the left sinus of Valsalva or inter-coronary commissure are observed less frequently than those situated above the right sinus. Tunnels feature a variable morphology and may access the left ventricle at some distance from the aortic valve. The aorto-ventricular tunnel will only traverse the intracardiac myocardium to access the left or right ventricular chamber in extremely rare cases [3].

Reports of the disease were first published by Edwards and Burchell in 1957 [4], describing the case of a child with a saccular aneurysm of the ascending aorta communicating with the left ventricle. The term “aortico-left ventricular tunnel” was coined by Levy and Coll. in 1963. This new entity was defined as an abnormal congenital communication between the root of the aorta and the left ventricle, bypassing the aortic valve and resulting in aortic regurgitation [5].

No links have been found between ALVT and any acknowledged genetic syndrome. Despite a lack of overall consensus in the field, in terms of embryology, ALVT likely stems from a combination of abnormal development of the endocardial cushions from which the pulmonary and aortic arteries are generated and subsequent abnormal separation of these roots [6].

Regarding ALVT pathophysiology, the vast majority of patients tend to develop heart failure during the first 12 months of life. However, the occurrence, degree, and progression of cardiac insufficiency varies ranging from rare cases characterised by many years of asymptomatic compensation to quick decompensation, sudden death, or intrauterine death. This variety may may be due to coronary artery compression and associated left or right ventricular outflow tract obstruction. Specifically, in ALVT with pulmonary stenosis, the onset of heart failure is delayed, whereas, in ALVT with associated aortic valve stenosis, congestive heart failure, with or without low cardiac output, occurs early [6].

ALVT features a prevalence corresponding just to 0.46% of all cardiac abnormalities identified by means of fetal echocardiography [2] and 0.05% among individuals undergoing cardiac catheterization [7]; ALVT is rarely detected in subjects of Asian or African descent [6].

In view of the rarity of ALVT, to date, only single case reports and small case series have been published. This is the first systematic review about ALVT, since no other previous relevant studies about this cardiac abnormality exist. As such, our study is covering an important research gap. This paper aims to provide a systematic review of the available English literature relating to ALVT and the clinical features, imaging diagnostic tools, treatment methods, and outcomes associated with this condition.

## 2. Methods: Search Strategy

A search of the electronic databases PubMed and Scopus was conducted ranging from date of inception up to February 16th 2024. The MeSH (Medical Subject Headings) search terms “case report” and/or “case series” and/or “aorta to left ventricular tunnel” and/or “aortico-left ventricular tunnel” and/or “aorto-left ventricular tunnel” were used. Animal studies, foetal studies (as such, the minimum age limit was 1 day of life), papers published in languages other than English and manuscripts failing to include at least seven of the nine analyzed features (age, sex, symptoms, electrocardiography, echocardiography, computed tomography (CT)/magnetic resonance imaging (MRI), association with other congenital heart disease, and outcome) were excluded. Due to the paucity of cases reported so far, it was impossible to set up other limitations, such as severity of symptoms.

### 2.1 Study Selection

According to the PRISMA approach, each author checked the shortlisted abstracts and judged whether they were appropriate. Full-texts were read when all the involved reviewers thought that the abstract might fulfill the previously chosen inclusion criteria.

Specifically, four reviewers were briefed for the identification of eligible studies (PPB, PC, PF, and MAP) by a fifth reviewer (MC) through successive stages of quadruplicate independent screening among selected titles and abstracts in groups of five, until a complete intra-examiner agreement was obtained (k scores from the first to the last calibration exercise: 0.79, 0.87, 0.93 and 1). A parallel, blind screening procedure of all titles and abstracts retrieved by the electronic search was performed by four reviewers (PPB, PC, PF, and MAP). The titles and abstracts were screened for subject importance. Studies that were not definitively excluded on the basis of abstract information were also selected for full-text screening. The reviewers examined the full text of all relevant studies to evaluate the possibility of inclusion. In the case of disagreement over study inclusion, a discussion was held with the fifth reviewer (MC) to reach an agreement.

### 2.2 Data Extraction

Data from the chosen single case reports and case series were extracted. The analysed features were: age at diagnosis, sex, clinical presentation, electrocardiography, imaging (echocardiography, CT scan and cardiac MRI), accompanying cardiac defects, and outcome.

### 2.3 Data Sharing

Data were introduced in the form of mean ± standard deviation. Mann-Whitney U tests were used to evaluate statistical significance when required. Univariate regression tests were performed on all variables, whilst multivariate logistic regression was only applied only on statistically significant variables using univariate analysis. Statistical significance was set to *p* < 0.05.

### 2.4 Study Selection Process

Overall, 559 potential single case reports or case series of ALVT were detected on PubMed. Ninety-nine were identical. A further 289 manuscripts were ruled out following a title check. The remaining 179 papers were subsequently evaluated in-depth. After screening, 109 papers were included in the analysis of patient characteristics and clinical outcome [7, 8, 9, 10, 11, 12, 13, 14, 15, 16, 17, 18, 19, 20, 21, 22, 23, 24, 25, 26, 27, 28, 29, 30, 31, 32, 33, 34, 35, 36, 37, 38, 39, 40, 41, 42, 43, 44, 45, 46, 47, 48, 49, 50, 51, 52, 53, 54, 55, 56, 57, 58, 59, 60, 61, 62, 63, 64, 65, 66, 67, 68, 69, 70, 71, 72, 73, 74, 75, 76, 77, 78, 79, 80, 81, 82, 83, 84, 85, 86, 87, 88, 89, 90, 91, 92, 93, 94, 95, 96, 97, 98, 99, 100, 101, 102, 103, 104, 105, 106, 107, 108, 109, 110, 111, 112, 113, 114, 115] (see **Supplementary Table 1**).

A PRISMA flow chart of the study selection process is showed in **Supplementary Table 2**.

## 3. Results

109 case reports or case series comprising a total of 177 patients were included. These papers represented all available cases published in English. However, largely due to the language exclusion criterion, not all published case reports were available for detailed clinical analysis. Although published in English, other reports were omitted based on non-availability of the publication from librarians or authors. One large case series was not included in the analysis due to a lack of detailed features of the 31 investigated patients [116].

**Fig. 1.**
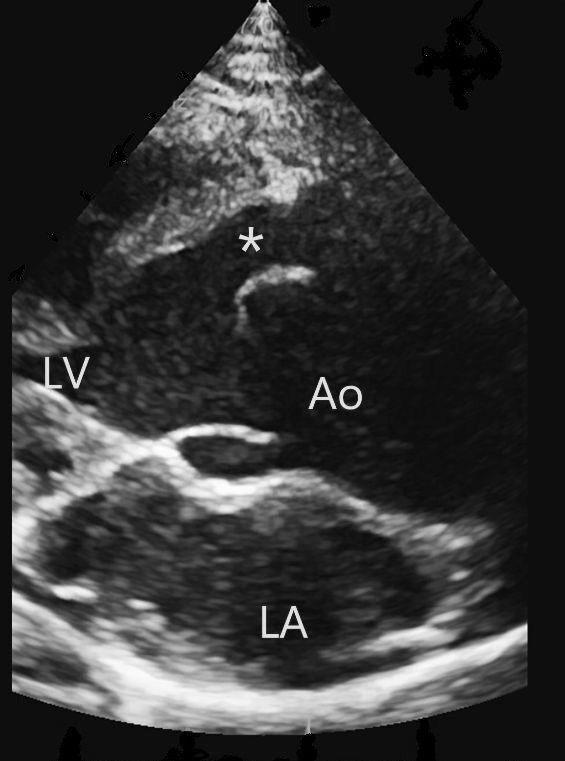
**Echocardiographic picture of ALVT**. Transthoracic parasternal long axis view showing an ALVT (*). Abbreviations: LV, left ventricle; Ao, aorta; LA, left atrium; ALVT, aorto-left ventricular tunnel.

Mean age at presentation was 9.5 ± 8.9 years, with a distinct male prevalence in disease distribution (64.2%, *p* < 0.02). However, 14.4% of cases provided no details of gender distribution. The most common clinical presentation was heart murmur (47.1%), followed by congestive heart failure (32.4%). Newborns and babies were found to be the category most affected by the latter condition, whilst heart murmur was observed predominantly in children and adults. Electrocardiography displayed left ventricular hypertrophy in 42% of cases observed. However, from the year 2000 onwards, electrocardiographic characteristics were rarely reported in ALVT case presentation, although 86.4% of cases were diagnosed by means of echocardiography (Fig. 1).

**Fig. 2.**
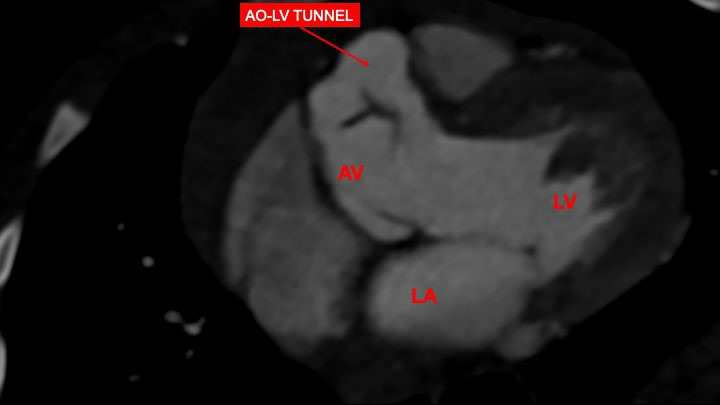
**Contrast-enhanced computed tomography**. This scan is particularly advantageous in providing an enhanced image of the tunnel. Abbreviations: AV, aortic valve; LV, left ventricle; LA, left atrium; AO, aortic valve.

**Fig. 3.**
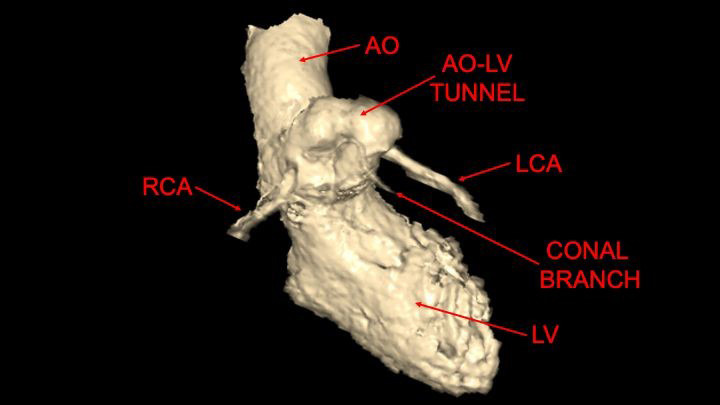
**Three dimensional computed tomography reconstruction**. This procedure enhanced clarification of the relationship between the tunnel and coronary arteries prior to intervention. Abbreviations: AO, aortic valve; LV, left ventricle; RCA, right coronary artery; LCA, left coronary artery.

The use of cardiac MRI and/or CT, first established in 2006, was applied in 17% of cases to confirm ALVT diagnosis. Contrast-enhanced CT (Figs. 2,3) in particular contributed significantly towards further outlining the tunnel and clarifying the connection to coronary arteries prior to intervention.

**Fig. 4.**
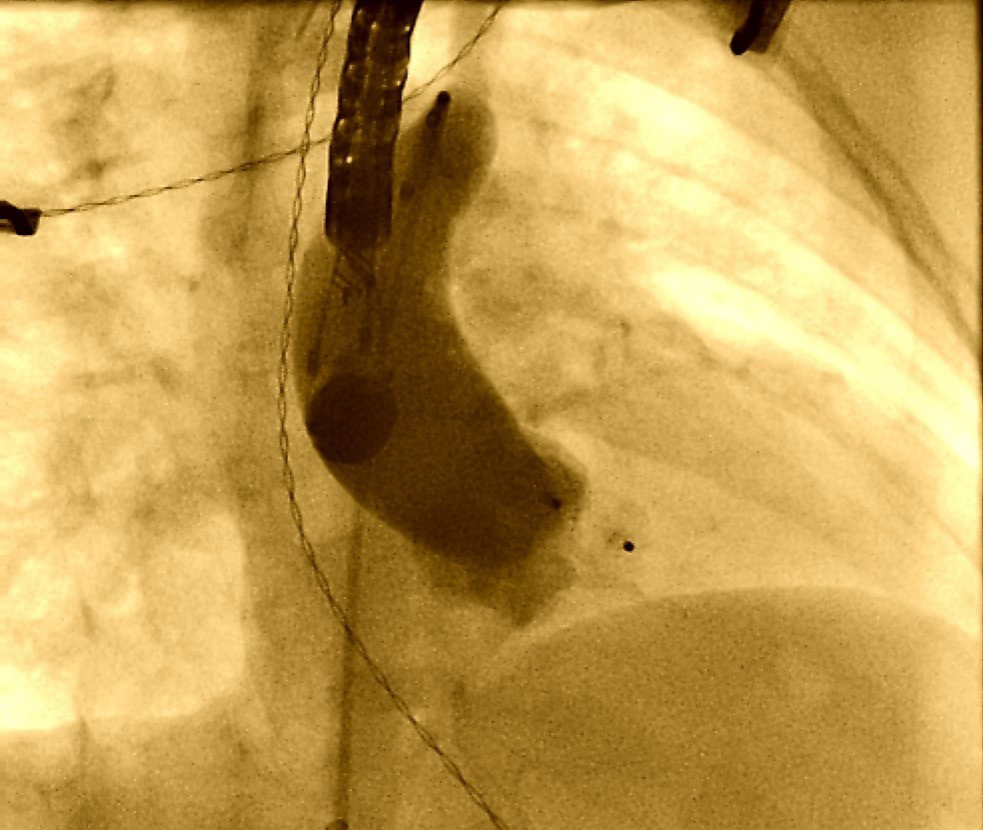
**Therapeutic cardiac catheterization**. Device-led procedure used in closure of ALVT. ALVT, aorto-left ventricular tunnel.

Associated cardiac abnormalities were detected in 39.8% of patients (unicuspid/bicuspid aortic valve with or without stenosis/atresia in 14.8%, coronary artery abnormalities 9.6%, left ventricular non compaction 3.4%). ALVT was treated surgically in 90.3% of cases, with selected cases (4.5%) being treated by means of catheter closure since the year 2000 (Fig. 4).

A unique case of spontaneous closure has also been reported [7]. The outcomes were largely favorable (death was reported in 5.7% of cases). Mild residual aortic regurgitation was present in 22.7% of the sample investigated.

In terms of statistics (univariate end multivariate), no independent risk factors for death were found (in all cases *p* = ns).

The main findings of the study are summarized in Table 1.

**Table 1.** **Isolated left ventricular apical hypoplasia patients’ features**.

Male-to-female ratio	1.79/1
Mean age at diagnosis	9.5 ± 8.9 years (range 1 day-64 years)
Symptoms	Asymptomatic with heart murmur (47.1%)
	Congestive heart failure (32.4%)
	Other (20.5%)
ECG changes	Left ventricular hypertrophy (42%)
Diagnosis	By echocardiography (86.4%)
	Adding cardiac magnetic resonance/computed tomography (17%)
Associated congenital heart disease	39.8%
Surgical treatment	90.3%
Device closure	4.5%
Post intervention aortic insufficiency	22.7%
Death	5.7%

Abbreviations: ECG, electrocardiogram.

## 4. Discussion

The true incidence of ALVT remains to be clarified [2]. It has been hypothesized to be in the range of 0.5% of all fetal cardiac malformations to less than 0.1% of congenitally abnormal hearts in clinico-pathological series [6]. Accordingly, although this study represents the largest systematic review in the field, it encompasses a mere 176 cases.

The embryologic origin of ALVT is still somewhat obscure, likely resulting from a combination of abnormal development of the cushions from which the aortic and pulmonary roots originate and subsequent atypical separation of these structures [6]. A series of other hypotheses have however been put forward with regard to the origin of ALVT. For instance, Levy *et al*. [5] hypothesized that ALVT may represent an abnormal coronary artery which opens into the left ventricle. Other hypotheses include a persistent fifth embryonic aortic arch resulting in impaired development of the distal bulbus cordis [117] or an intrauterine ruptured sinus of Valsalva aneurysm that evolves into a channel of communication with the myocardial sinusoids and, subsequently, the ventricle [118]. As confirmed in our analysis, no specific genetic defect responsible for this process has yet been identified [6].

An anatomical classification of ALVT has been proposed by Hovaguimian and Coll., namely:

- Type 1: the abnormality comprises a simple tunnel with a slit-like opening at the aortic end, accompanied by an absence of any aortic valve distortion;

- Type 2: presence of a large extracardiac aortic wall aneurysm of the tunnel featuring an oval opening at the aortic end, with or without aortic valve distortion;

- Type 3: an intracardiac aneurysm of the septal portion of the tunnel, with or without right ventricular outflow tract obstruction;

- Type 4: a combination of types 2 and 3 [119].

The frequently observed aneurysmal size and shape of the tunnel should be highlighted [18].

The above-stated nomenclature is still in place today, although an attempt to review this was made by members of the Society of Thoracic Surgeons (STS)-Congenital Heart Surgery Database Committee and representatives of the European Association for Cardiothoracic Surgery. Nevertheless, the Hovaguimian classification continues to be considered a highly beneficial tool by surgeons in the field [120].

In line with previous reports, the findings of this systematic review indicated a high male prevalence in distribution of the disease [121].

In view of its accuracy in describing the type and involvement of the cardiac structure, echocardiography is the preferred technique for use in diagnosing non-invasive ALVT [90]. Indeed, echocardiographic findings highlight the presence of ventricular septal dropout immediately below the aortic valve. Further evaluation -adopting a parasternal long-axis view- provides evidence of a rim of tissue extending in front of and behind the furthest cephalad section of the ventricular septum and accessing the aortic root marginally distal to the aortic valve and right coronary artery. The left ventricle is directly linked to the ascending aorta, distal to the aortic valve, through a paravalvular tunnel. The latter is usually aneurysmatic. Flow through the tunnel is forward in systole and retrograde from the aorta to the left ventricle in diastole. This results in a cardiac to-and-fro murmur which, as highlighted in this review, is the most common clinical manifestation of the disease, particularly in children and adult patients [5, 122]. The resulting turbulence in the aortic root may produce progressive aortic valvular damage leading to consequent heart failure. Sudden death has also been reported [117, 123]. Should the tunnel and link to the proximal section of the ascending aorta not be sufficiently visualized, it may prove difficult to distinguish dropout of the ventricular septum from a ventricular septal defect. However, a right-to-left ventricular shunt is absent at birth (or left-to-right at an older age) [22].

ALVT originates distal to the aortic sinuses, thus distinguishing the malformation from a ruptured congenital sinus of a Valsalva aneurysm. On the other hand, the absence of any relationship between this tunnel and the right coronary ostium rules out the possibility of the condition being a right coronary artery-left ventricle communication [123, 124].

Despite the routine efficacy of transthoracic echocardiography in ALVT diagnosis, a misdiagnosis rate of up to 17.1% is reported [90].

Echocardiography was frequently adopted in the past in combination with cardiac catheterization to clarify ALVT anatomy, origin of the coronary arteries or associated congenital heart defects [6]. Prior to undertaking surgery for ALVT, a concerted effort should be made to verify the anatomy of the coronary artery, as failure to do so in the case of the aberrant origin of a coronary artery may hamper successful surgical correction [125]. During cardiac catheterization, the contrast introduced into the left ventricle fills the aorta through two separate channels: one via the aortic valve and the other via a separate tunnel arising anteriorly and superiorly from the left ventricle and communicating with the ascending aorta, passing around the aortic valve [16]. However, cardiac catheterization has been progressively replaced by CT and/or cardiac MRI [126, 127].

Antenatal diagnosis by fetal echocardiography combined with color flow Doppler is feasible after 18 weeks’ gestation [2]. Cases diagnosed in utero are usually more severe and feature a worse outcome. In a retrospective, two-center review of all cases of ALVT diagnosed in utero from 1983 to 1995, three cases were detected using Doppler echocardiography between 22 and 24 weeks’ gestation. Prenatal ALVT was associated with severe left ventricular dysfunction, aortic valve abnormalities, fetal hydrops, and dramatic outcome: one death occurred in utero, another immediately after birth, and in the third case, the pregnancy was terminated. In all three cases, ALVT diagnosis was confirmed by autopsy [128]. The first report of an ALVT diagnosed in utero with a favorable outcome after surgery at three months of age was reported in 2000 [44].

Spontaneous closure of ALVT was only observed in exceedingly rare, asymptomatic patients with a small tunnel [7]. However, generally speaking, prompt surgical repair is usually required [6]. Surgery should be considered early in life, with the chosen technique aimed at consolidating the aortic annulus without deformation and closing the aorta-ventricular window [129]. Surgical closure of ALVT is recommended at the time of diagnosis, even in asymptomatic patients, based on the inadequacy of medical intervention, risk of developing severe aortic regurgitation and obtaining of good surgical results. Surgical intervention is aimed at obliterating the tunnel. A series of techniques are available for use, including: (a) closure of the aortic orifice of the tunnel by direct suture (continuous, interrupted or single or double layer) or by means of a patch (Dacron, pericardium, Teflon); (b) closure of the ventricular end of the tunnel; (c) obliteration of the tunnel (i.e., ligation of the tunnel, partial resection of the tunnel, or filling of the tunnel with gel-foam); (d) obliteration of both orifices (aortic and ventricular) [130, 131]. It has been suggested that by closing the aortic defect by means of direct suture, distortion of the aortic cusps may ensue, pulling them toward the weak aortic wall, which remains unsupported within the dilated aortic sinus. Accordingly, aortic regurgitation may persist and progress despite ALVT repair in infancy. From this perspective, the patch technique is believed to reduce this risk by reducing distorting of the cusps to a minimum, thus potentially resulting in a lower risk of aortic regurgitation [54].

A significant number of patients present with an aneurysmatic tunnel, with special support of the aortic root in relation to the right aortic sinus recommended as an important element in surgical correction [132]. The “tunnel” is of course not an actual tunnel, but rather a localized aperture at the level of access to the right coronary cusp. Following closure of the tunnel, aortic root dilatation results in a poorly supported aortic valve and weak root. Initially therefore, the ALVT should be closed surgically, whilst at the same time aiming to potentiate and support the right aortic sinus in order to preserve cusp competence [131].

It would be reasonable to determine the aortic sinus as the point at which the ALVT orifice is located, particularly as, in cases featuring location of the orifice at the right aortic sinus, the ALVT passes in front of the ascending aorta, thus facilitating a surgical approach through the tunnel wall. Conversely, when the left aortic sinus is determined as the location of the orifice, the ALVT passes behind and to the side of the ascending aorta. Similar cases are extremely rare and may complicate resection of the tunnel wall, thus indicating aortotomy as a preferred option in the surgical repair of ALVT [133]. Particular care should be taken when the left aortic sinus produces the aortic orifice, in order to prevent injury to the left coronary artery during surgical repair [134].

Surgery undoubtedly represents the gold standard for treatment; however, in selected anatomically-suitable cases, transcatheter closure of ALVT may be considered. The latter approach was first reported by Chessa *et al*. [43] in the year 2000 using an Amplatzer patent ductus arteriosus (PDA) occluder device (AGA Medical Corporation, Golden Valley, Minnesota) to obliterate the tunnel.

All treated patients require life-long follow-up for recurrence of ALVT, aortic valve regurgitation, left ventricular dysfunction, and aneurysmal dilatation of the ascending aorta [6]. Following surgical closure of AVLT, the most commonly observed complication is undeniably aortic regurgitation caused as the result of extreme dilatation of the ascending aorta and aortic ring with a hanging right aortic cusp. The intrinsic nature of the lesion is indicated by detachment of the right or left coronary aortic leaflets from the aortic root. This detachment withdraws support for either the right or left coronary aortic leaflet, thus resulting in progressive aortic regurgitation [3] which, at times, require aortic valve replacement [135]. Post-treatment aortic incompetence had previously been reported in 67% of patients [130], although the updated findings shared in this review reduced this percentage to 22.7%. Prompt surgical correction is indicated to prevent progression of damage to the aortic valve, potentially leading to approx. half of all patients requiring replacement of the aortic valve replacement at some point in the future [130].

Lesions of the aortic valve, mainly in the form of bicuspid valve with or without obstruction, were detected in 14.8% of patients, and coronary artery abnormalities in 9.6%. These conditions had been highlighted in 20% and 45% of patients, respectively, in previous outdated reports [39, 61].

One case of left ventricular outflow tract aneurysm mimicking an ALVT detected during surgery, has also been reported [136]. An anecdotal case of stroke due to ALVT was described in 2011. Turbulent blood flow in the region of the tortuous tunnel with a stenotic component and low flow areas were likely the cause of cardiogenic cerebral emboli [69].

Nowadays, the mortality rate for ALVT (5.7%) is however slightly lower than previously reported (7.14%) [121].

The present study certainly has its own limitations. Firstly, the small sample size due to the rarity of ALVT cases. With just 8 cases treated by catheter intervention, a comparison with surgery was not possible. The reduced sample size may have also affected statistical analysis. Secondly, the retrospective study design (inferior level of evidence compared with prospective studies; cases often not representative of the general population and prone to selection bias. In fact, the quality and heterogeneity of the included literature might affect the generalizability of the results). Lastly, unfortunately there is no previous robust literature in the field, only single case reports and limited case series. As such, no comparative analysis is possible. This is the first systematic review in the field encompassing as many cases as possible.

## 5. Conclusions

To summarize, ALVT is an extremely rare congenital cardiac abnormality of moderate complexity [137] diagnosed in both newborns and adult patients. Diagnosis is usually based on echocardiographic findings subsequently refined by catheterization, CT or MRI. Surgery should be undertaken as soon as possible following diagnosis due to the inefficacy of medical treatment. In selected cases, transcatheter closure of ALVT may represent a valid option. Mortality rates, with or without intervention, are by no means trivial. Although to date no surgical techniques have proved their superiority in preventing late-onset complications, other complications, including, in particular, residual aortic valve regurgitation, may also influence post-intervention prognosis. In view of the rarity of the disease, the setting-up of an International Registry is recommended to gather more data on ALVT or studying the long-term outcomes of different therapeutic approaches [138, 139].

## Availability of Data and Materials

All datasets on which the conclusions of a manuscript depend are shared in the **Supplementary Table 1**.

## Supplementary Material

Supplementary material associated with this article can be found, in the online version, at https://doi.org/10.31083/RCM26005.
