# Supplementary material for: Aorto-Left Ventricular Tunnel: The First Systematic Review of An Uncommon Entity (177 Worldwide Cases from 1965 to 2024)
Source: Rev Cardiovasc Med. 2025 Feb 20;26(2):26005. doi: 10.31083/RCM26005 (PMC11868909; doi:10.31083/RCM26005)
Supplement: Supplementary file 1 [file 2153-8174-26-2-26005-s1.zip › Supplementary Material.docx]

**Table 1.** List of the case reports and case series which have been published in the field.

| **Author** | **Patient’s age (years)** | **Patient’s gender** | **Clinical presentation** | **Electrocardiogram** | **Diagnosis by echocardiography** | **Diagnosis by CT/MRI** | **Associated congenital cardiac abnormalities** | **Outcome** |
| --- | --- | --- | --- | --- | --- | --- | --- | --- |
| Cooley RN, et al. **[8]** | 16 months | Male | Exertional dyspnoea | LVH | No | No | Aortic stenosis | Death |
| Fishbone G, et al. **[9]** | 11 | Female | Heart murmur | LVH | No | No |  | Successful surgery. Residual mild aortic regurgitation |
| Pérez-Martínez V, et al. **[10]** | 10 months | Male | Dyspnoea | LVH | No | No | No | Death |
| Bizouati G, et al. **[11]** | 20 | Male | Heart failure due to aortic valve regurgitation | Intermittent left bundle branch block | No | No | No | Successful surgery |
| Mair DD, et al. **[12]** | 9 months | Male | Heart failure due to aortic valve regurgitation | None | No | No | No | Successful surgery |
| Neches WH, et al. **[13]** | 4 | Male | Heart failure due to aortic valve regurgitation | Normal | No | No | DORV | Successful surgery |
| Nichols GM, et al. **[14]** | 5 months | Female | Asymptomatic | LVH with ST-T wave changes | No | No | No | Successful surgery. Residual mild aortic regurgitation |
| Giardina AC, et al. **[15]** | 1 day | Female | Severe congestive heart failure | LVH with ST-T wave changes and rightward deviation | No | No | ASD, PDA | Ventricular tachycardia and death |
| Sung CS, et al. **[16]** | 7 months | Male | Heart murmur assessment | LVH | No | No |  | Successful surgery. Residual mild aortic regurgitation |
| Villani M, et al. **[17]** | 28 months | Not reported | Moderate congestive heart failure | LVH | No | No | No | Successful surgery. Residual mild aortic regurgitation |
|  | 3 months | Not reported | Severe congestive heart failure | LVH | No | No | Mitral regurgitation | Ventricular fibrillation and death |
| Saylam A, et al. **[18]** | 10 | Male | Heart murmur | LVH | No | No | No | Successful surgery |
| Ruschewski W, et al. **[19]** | 4 | Male | Heart murmur | LVH | No | No | No | Re-do surgery for suture dehiscence |
|  | 5 | Male | Heart murmur | LVH | No | No | No | Successful surgery |
|  | 6 | Male | Heart murmur | LVH | No | No | No | Successful surgery |
|  | 14 | Male | Heart murmur | LVH |  |  | Aneurysm of the membranous portion of the interventricular septum | Re-do surgery |
|  | 11 | Male | Dyspnoea on exertion, reduced exercise tolerance | Normal | No | No | No | Successful surgery |
| Turley K, et al. **[20]** | 1 day | Male | Respiratory distress | Right atrial enlargement, then LVH | Yes | No | PS, BAV | Successful surgery |
| Björk VO, et al. **[21]** | 7 days | Female | Severe congestive heart failure | Severe left ventricular strain with large R-waves and inverted T-waves. The ST-segments were depressed | Yes | No | No | Successful surgery |
| Fripp RR, et al. **[22]** | 1 day | Not reported | Severe congestive heart failure | Normal | Yes | No | BAV | Successful surgery |
| Bash SE, et al. **[23]** | 7 months | Male | Severe congestive heart failure | LVH | Yes | No | VSD | Complete heart block and death |
| Grant P, et al. **[24]** | 2 | Female | Heart murmur | LVH | Yes | No | No | Successful surgery |
| Guyton RA, et al. **[25]** | 3 days | Male | Congestive heart failure | LVH | Yes | No | Aortic atresia | Re-do surgery |
| Lindberg H, et al. **[26]** | 5 months | Female | Heart murmur and cardiomegaly on chest X-ray | LVH | Yes | No | No | Successful surgery. Residual mild aortic insufficiency |
| Hucin B, et al. **[27]** | 1 day | Male | Congestive heart failure | LVH with strain and ischaemia | Yes | No | VSD, PDA | Successful surgery |
|  | 1 day | Male | Cyanosis, then congestive heart failure | LVH | Yes | No | PDA | Successful surgery |
| Guo DW. **[28]** | 5 | Female | Dyspnoea and palpitations | Not reported | Yes | No | No | Successful surgery |
| Kafka H, et al. **[29]** | 22 | Male | Asymptomatic | Normal | Yes | No | No | Not treated |
| Horváth P, et al. **[30]** | 6 days | Not reported | Congestive heart failure | LVH | Yes | No | PDA | Operative death |
|  | 17 months | Not reported | Heart murmur | LVH | Yes | No | No | Successful surgery. Residual moderate aortic regurgitation |
|  | 16 months | Not reported | Heart murmur | LVH | Yes | No | No | Successful surgery |
|  | 15 months | Not reported | Heart murmur | LVH | Yes | No | No | Successful surgery |
|  | 11 | Not reported | Heart murmur | LVH | Yes | No | No | Successful surgery |
|  | 6 | Not reported | Heart murmur | LVH | Yes | No | BAV | Successful surgery |
|  | 3 months | Not reported | Heart murmur | LVH | Yes | No | PDA | Successful surgery |
|  | 4 months | Not reported | Heart murmur | LVH | Yes | No | No | Successful surgery. Residual mild aortic regurgitation |
|  | 6 hours | Not reported | Congestive heart failure | LVH | Yes | No | VSD, PDA, origin of the left coronary artery from the tunnel | Successful surgery. Residual mild aortic regurgitation |
|  | 14 hours | Not reported | Congestive heart failure | LVH | Yes | No | PDA | Successful surgery |
|  | 23 days |  | Heart murmur | LVH | Yes | No | PDA, BAV, origin of the right coronary artery from the tunnel | Successful surgery |
|  | 6 | Not reported | Heart murmur | Normal | Yes | No | No | Successful surgery |
|  | 9 days | Not reported | Congestive heart failure | LVH | Yes | No | No | Successful surgery |
| Sreeram N, et al. **[31]** | 5 days | Female | Heart murmur, then congestive heart failure | LVH | Yes | No | No | Successful surgery |
| Sreeram N, et al. **[32]** | 10 months | Male | Heart murmur | LVH | Yes | No | VSD | Successful surgery. Residual mild aortic regurgitation |
|  | 22 months | Female | Heart murmur | Normal | Yes | No | No | Successful surgery. Residual mild aortic regurgitation |
|  | 2 months | Male | Heart murmur | LVH | Yes | No | No | Successful surgery. Residual mild aortic regurgitation |
|  | 5 days | Male | Heart murmur, then congestive heart failure | LVH | Yes | No | No | Successful surgery |
| Webber S, et al. **[33]** | 1 day | Male | Congestive heart failure | Bi-atrial hypertrophy, LVH | Yes | No | BAV (critical aortic stenosis) | Successful surgery |
| Knott-Craig CJ, et al. **[34]** | 5 | Male | Mild dyspnoea | LVH with overload | Yes | No | RVOTO caused by the aneurysmal dilatation of the tunnel | Successful surgery |
| Bitar FF, et al. **[35]** | 1 day | Male | Heart murmur, mild cyanosis | LVH with strain pattern | Yes | No | Atretic aortic valve, PDA | Successful surgery. Residual moderate aortic regurgitation |
| Chen YF, et al. **[36]** | 4 months | Male | Congestive heart failure | LVH | Yes | No | No | Successful surgery |
| Kakadekar AP, et al. **[37]** | 2 days | Male | Congestive heart failure | Not reported | Yes | No | No | Redo-surgery after 10 years because of residual ALVT and severe aortic regurgitation |
| Weldner P, et al. **[38]** | 2 weeks | Female | Heart murmur | LVH | Yes | No | Dysplastic aortic valve, PS | Successful surgery (moderate PS was not treated) |
| Rauzier JM, et al. **[39]** | 6 weeks | Male | Congestive heart failure | LVH and ischaemia | Yes | No | Right coronary atresia | Successful surgery |
| Michielon G, et al. **[40]** | 11 | Male | Heart murmur | LVH | Yes | No | No | Successful surgery |
| Grünenfelder J, et al. **[41]** | 14 months | Male | Heart murmur | Not reported | Yes | No | Origin of the right coronary artery from the tunnel | Successful surgery |
| Parra Bravo JR, et al. **[42]** | 1 month | Male | Congestive heart failure | LVH | Yes | No | No | Successful surgery |
|  | 9 | Male | Heart murmur | LVH | Yes (not diagnostic) | No | No | Successful surgery. Residual mild aortic regurgitation |
|  | 11 | Female | Heart murmur | LVH | Yes | No | No | Successful surgery. Residual mild aortic regurgitation |
|  | 14 | Female | Heart murmur | LVH | Yes (not diagnostic) | No | No | Successful surgery. Residual mild aortic regurgitation |
| Chessa M, et al. **[43]** | 14 | Male | Heart murmur | Not reported | Yes | No | No | Successful interventional procedure |
| Grab D, et al. **[44]** | 1 day | Male | Heart murmur at birth, then congestive heart failure | Not reported | Yes (foetal echo) | No | No | Successful surgery (at 3 months) |
| Kuo HC, et al. **[45]** | 3 months | Male | Congestive heart failure | LVH | Yes | No | Single coronary artery | Successful surgery |
| Ando M, et al. **[46]** | 31 | Male | Heart murmur | Normal | Yes | No | No | Successful surgery |
| Martins JD, et al. **[47]** | 1 day | Male | Congestive heart failure | Not reported | Yes | No | BAV with critical stenosis, muscular VSD, single left coronary artery | Successful surgery |
|  | 4 | Male | Heart murmur | Not reported | Yes | No | Single left coronary artery | Successful surgery. Residual Mild aortic regurgitation |
|  | 15 days | Female | Congestive heart failure | Not reported | Yes | No | Right coronary artery to distal tunnel fistula | Successful surgery |
|  | 15 days | Male | Congestive heart failure | Not reported | Yes | No | No | Successful surgery |
|  | 5 months | Female | Heart murmur | Not reported | Yes | No | No | Successful surgery. Residual mild aortic regurgitation |
|  | 2 months | Male | Heart murmur | Not reported | Yes | No | No | Successful surgery |
|  | 3 months | Female | Congestive heart failure | Not reported | Yes | No | BAV | Surgery. Residual moderate ALVT which has been closed by means of a coil |
|  | 3 | Male | Heart murmur | Not reported | Yes | No | No | Successful surgery |
|  | 1 day | Female | Congestive heart failure | Not reported | Yes | No | Unicommussural aortic valve with critical stenosis, subaortic stenosis | Successful surgery. Residual mild aortic regurgitation |
|  | 3 | Male | Heart murmur | Not reported | Yes | No | No | Successful surgery |
|  | 4 days | Male | Congestive heart failure | Not reported | Yes | No | No | Spontaneous closure |
| Vida VL, et al. **[48]** | 4 | Not reported | Asymptomatic | Not reported | Yes | No | BAV | Successful surgery |
| Kolcz J, et al. **[49]** | 1 day | Male | Respiratory distress | Not reported | Yes | No | No | Successful surgery |
| Siepe M, et al. **[50]** | 1 day | Not reported | Congestive heart failure | Not reported | Yes | No | Aortic atresia | Successful surgery. Residual mild aortic regurgitation |
| Wang JK, et al. **[51]** | 2 | Male | Not reported | Not reported | Yes | No | No | Surgery. Transcatheter closure of residual ALVT at the age of 17. |
| Sakurai M, et al. **[52]** | 3 | Male | Heart murmur | LVH with overload | Yes | No | No | Surgery. Aortic valve replacement at the age of 10. Ascending aorta aneurysm and replacement at the age of 18 |
| Honjo O, et al. **[53]** | 4 | Female | Heart murmur | Not reported | Yes | No | No | Successful surgery |
|  | 4 months | Male | Heart murmur | LVH | Yes | No | No | Successful surgery. Residual mild aortic regurgitation |
| Sadeghpour A, et al. **[54]** | 26 | Female | Heart murmur, dyspnoea | Not reported | Yes | No | LVNC | Not reported |
| Mitropoulos FA, et al. **[55]** | 10 days | Male | Heart murmur | Not reported | Yes | No | PDA | Successful surgery |
| Coskun KO, et al. **[56]** | 45 | Male | Unstable angina pectoris | LVH | Yes | Yes | No | Recurrence of ALVT operated at the age of 30. Successful surgery |
| Kenny D, et al. **[57]** | 1 day | Male | Congestive heart failure | Not reported | Yes | No | No | Successful surgery |
| Chehab G, et al. **[58]** | 2 months | Male | Heart murmur | Not reported | Yes | No | No | Successful surgery. Residual mild aortic regurgitation |
| Ono M, et al. **[59]** | 10 months | Male | Heart murmur | Not reported | Yes | No | No | Successful surgery |
| Henaine R, et al. **[60]** | 1 day | Not reported | Heart murmur | Left axis deviation | Yes | No | No | Successful surgery |
| Kondrachuk O, et al. **[61]** | 3 days | Male | Congestive heart failure | Not reported | Yes | Yes | No | Successful surgery |
| Schreiber C, et al. **[62]** | 1 day | Female | Congestive heart failure | Not reported | Yes | No | Dysplastic aortic valve with critical stenosis | Successful surgery |
| Li D, et al. **[63]** | 32 | Male | Congestive heart failure | LVH, first degree AV block | Yes | No | No | Successful surgery |
|  | 18 | Male | Heart murmur | LVH | Yes | Yes | No | Successful surgery |
|  | 39 | Male | Congestive heart failure | First degree AV block | Yes | No | Dissecting aortic aneurysm | Surgery. AV perivalvular leak |
|  | 4 | Male | Heart murmur | LVH | Yes | Yes | BAV | Successful surgery |
|  | 3 | Male | Congestive heart failure | LVH | Yes | Yes | No | Successful surgery |
|  | 42 | Male | Congestive heart failure | LVH | Yes | No | No | Successful surgery |
|  | 5 | Male | Congestive heart failure | LVH, first degree AV block, PVCs | Yes | No | BAV | Death before surgery |
|  | 58 | Male | Congestive heart failure | LVH | Yes | No | No | Successful surgery |
|  | 9 months | Female | Heart murmur | LVH | Yes | No | Ventricular septal aneurysm | Successful surgery |
| Troubil M, et al. **[64]** | 30 | Male | Heart murmur | LVH | Yes | No | PDA | Successful surgery |
| Bautista_Hernandez V, et al. **[65]** | 2 days | Not reported | Heart murmur | Not reported | Yes | No | Right coronary artery arising from tunnel | Successful surgery |
| Nezafati MH, et al. **[66]** | 3 | Male | Heart murmur | Not reported | Yes | No | No | Successful surgery |
| Kaiser CA, et al. **[67]** | 4 | Male | Heart murmur | Not reported | Yes | Yes | No | Successful surgery |
| Saritas T, et al. **[68]** | 7 months | Male | Congestive heart failure | Not reported | Yes | No | No | Successful surgery. Residual mild aortic regurgitation |
|  | 10 | Male | Heart murmur | Not reported | Yes | No | No | Unsuccessful transcatheter closure. Successful surgery. Residual mild aortic regurgitation |
|  | 1,5 months | Male | Congestive heart failure | Not reported | Yes | No | No | Successful surgery |
| Singh A, et al. **[69]** | 1 day | Male | Congestive heart failure | Not reported | Yes | No | No | Successful surgery |
|  | 1 day | Female | Congestive heart failure | Not reported | Yes | No | No | Death after surgery due to multiorgan failure |
|  | 2 days | Male | Heart murmur | Transient ECG changes due to kinking of a coronary artery, which was managed conservatively | Yes | No | No | Successful surgery |
|  | 1 day | Male | Congestive heart failure | Not reported | Yes | No | BAV | Successful surgery |
| Wollenweber FA, et al. **[70]** | 42 | Male | Stroke | Sinus rhythm | Yes | No | No | Successful surgery |
| Colak N, et al. **[71]** | 18 | Male | Dyspnoea on exertion | Not reported | Yes | Yes | Single coronary artery | Residual ALVT after surgery at the age of 17. Successful re-do surgery |
| Zhang YL, et al. **[72]** | 39 | Male | Dyspnoea on exertion | Paroxysmal junctional tachycardia and complete left bundle branch block | Yes | No | Severe mitral regurgitation | Successful surgery |
| Mueller C, et al. **[73]** | 17 days | Male | Congestive heart failure | Not reported | Yes | No | No | Successful surgery |
| Pockett CR, et al. **[74]** | 1 day | Female | Congestive heart failure | Not reported | Yes | No | Unicuspid aortic valve, PS, hypoplastic right ventricle, muscular VSD, PDA | Heart transplant at 45 days, then death. |
| Furtado AD, et al. **[75]** | 16 | Female | Heart murmur | Not reported | Yes | Yes | No | Successful surgery |
| Thomas E, et al. **[76]** | 4.5 | Male | Heart murmur | Normal | Yes | No | No | Transcatheter closure |
| Malakan Rad E, et al. **[77]** | 2 days | Male | Heart murmur | LVH | Yes | No | LVNC, right coronary artery arising from tunnel, PDA | Successful surgery |
| Shiraishi S, et al, **[78]** | 1 | Female | Heart murmur | Not reported | No | Yes | No | Successful surgery |
|  | 15 | Female | Dyspnoea on exertion | Not reported | Yes | Yes | No | Successful surgery. Residual mild aortic regurgitation |
| Yildirim A, et al. **[79]** | 11 | Male | Heart murmur, then congestive heart failure | Sinus tachycardia, negative T wave in leads V4-V6 | Yes | No | Aortic aneurysm | Successful surgery |
| Horinouchi T, et al. **[80]** | 1 day | Male | Congestive heart failure | Not reported | Yes | No | No | Successful surgery. Residual mild aortic regurgitation |
| Paech C, et al. **[81]** | 1 day | Not reported | Heart murmur | Not reported | Yes | No | Dysplastic aortic valve with severe stenosis | Successful surgery (ALVT closure and Ross procedure) |
| Jone PN, et al. **[82]** | 1 day | Male | Heart murmur | Sinus tachycardia | Yes | No | BAV, dilated ascending aorta | Successful surgery |
| Cebeci M, et al. **[83]** | 33 | Female | Dyspnoea on exertion | Not reported | Yes | No | No | Successful surgery |
| Song L, et al. **[84]** | 2 | Male | Heart murmur | Not reported | Yes | Yes | Left coronary artery abnormality | Successful surgery |
| Zhu J, et al. **[85]** | 2 months | Male | Congestive heart failure | Not reported | Yes | No | VSD | Successful surgery |
| Xia H, et al. **[86]** | 41 | Male | Palpitations, dyspnoea, heart murmur | CHB | Yes | No | No | Infective endocarditis. Death |
|  | 37 | Female | Palpitations, heart murmur | LVH | Yes | No | Left coronary artery anomaly | Successful surgery. Residual aortic regurgitation |
|  | 40 | Male | Heart murmur | LVH | Yes | No | No | Successful surgery. Residual mild aortic regurgitation |
|  | 25 | Male | Heart murmur | LVH | Yes | No | No | Successful surgery. Residual mild aortic regurgitation |
|  | 34 | Male | Heart murmur | LVH | Yes | No | No | Successful surgery. Residual aortic regurgitation |
|  | 42 | Male | Palpitations, dyspnoea, heart murmur | NSVT | Yes | No | No | Successful surgery |
|  | 36 | Male | Palpitations, dyspnoea, heart murmur | LVH | Yes | No | No | Successful surgery. Residual aortic regurgitation |
|  | 20 | Female | Heart murmur | Normal | Yes | No | No | Successful surgery |
|  | 29 | Male | Palpitations, dyspnoea, heart murmur | LVH | Yes | No | No | Successful surgery. Residual aortic regurgitation |
| Kharwar RB, et al. **[87]** | 25 | Male | Syncope | CHB | Yes | Yes | Multi-aneurysmal heart. Infundibular PS | Permanent pacemaker insertion. Successful surgery |
| Smith BM, et al. **[88]** | 1 day | Not reported | Congestive heart failure | Not reported | Yes | No | Right coronary artery arising from the tunnel, LVNC | Successful surgery. Residual mild aortic regurgitation |
|  | 14 | Not reported | Asymptomatic | Not reported | Yes | No | Aortic aneurysm, LVNC | Successful surgery |
|  | 1 day | Not reported | Congestive heart failure | Not reported | Yes | No | LVNC | Successful surgery |
|  | 3 days | Not reported | Congestive heart failure | Not reported | Yes | No | Severe aortic stenosis, LVNC | Heart transplantation |
| Hartyánszky I, et al. **[89]** | 14 | Male | Heart murmur | Not reported | Yes | Yes | No | Successful surgery |
| Nakamura Y, et al. **[90]** | 1 day | Male | Transient circulatory collapse | Not reported | Yes | No | PDA | Successful surgery |
| Xie M, et al. **[91]** | 21 | Male | Dyspnoea | Not reported | Yes | No | VSD | Surgery. Residual ALVT |
|  | 40 | Male | Asymptomatic | Not reported | Yes | No | BAV with aortic stenosis | Successful surgery. Residual mild aortic regurgitation |
|  | 31 | Female | Dyspnoea | Not reported | Yes | No | No | Successful surgery |
|  | 3 | Male | Asymptomatic | Not reported | Yes | Yes | No | Successful surgery. Residual mild aortic regurgitation |
|  | 41 | Male | Dyspnoea | Not reported | Yes | No | No | Successful surgery |
|  | 12 | Female | Asymptomatic | Not reported | Yes | No | No | Successful surgery |
| Maghrabi K, et al. **[92]** | 4 days | Male | Heart murmur | Not reported | Yes | No | No | Successful surgery |
| Djukic M, et al. **[93]** | 2 months | Male | Heart murmur | Normal | Yes | No | No | Surgery. Re-do surgery for residual ALVT at the age of 7. Residual mild aortic regurgitation |
| Rao YM, et al. **[94]** | 5 | Male | Dyspnoea, heart murmur | Not reported | Yes | No | No | Successful surgery |
| Hadeed K, et al. **[95]** | 7 days | Not reported | Heart murmur, feeding difficulties | Not reported | Yes | Yes | No | Successful surgery |
| Portelli et al. **[96]** | 6 | Male | Not reported | Not reported | No | No | Aortic root aneurysm | Aortic root replacement at the age of 55 |
| Kosutic J, et al. **[97]** | 1 day | Female | Congestive heart failure | Not reported | Yes | No | BAV, PDA | Death |
| Khajali Z, et al. **[98]** | 20 | Female | Palpitations | LVH and strain pattern in the inferior and precordial leads | Yes | Yes | Dilated aortic root | Successful surgery |
| Perrier SL, et al. **[99]** | 3 weeks | Male | Heart murmur | LVH with strain pattern and inferolateral T wave inversion | Yes | No | Right coronary artery arising from the tunnel | Successful surgery |
| Bakas AM, et al. **[100]** | 4 | Female | Heart murmur | Not reported | Yes | No | Perimembranous VSD | Successful surgery |
| Chen PC, et al. **[101]** | 1 month | Male | Heart murmur | Not reported | Yes | Yes | Large muscular VSD, right coronary artery atresia, right ventricular sinusoids | Successful surgery |
| Wu W, et al. **[102]** | 31 | Male | Dyspnoea | Not reported | Yes | Yes | No | Surgery. Outcome not reported |
| Ma X, et al. **[103]** | 5 | Female | Asymptomatic | Not reported | Yes | Yes | BAV, anomalous origin of the right coronary artery | Successful surgery |
| Alpat S, et al. **[104]** | 2 | Male | Not reported | Not reported | Yes | No | Dilated aortic root | Re-do surgery for aortic root replacement at the age of 32 |
| Jhou HJ, et al. **[105]** | 4.5 months | Male | Congestive heart failure | Not reported | Yes | No | No | Surgery. Re-do surgery for residual ALVT and aortic valve replacement at the age of 18. Aortic root replacement at the age of 27. |
| Chen LL, et al. **[106]** | 58 | Male | Chest tightness | LVH | Yes | Yes | BAV with severe stenosis | Successful surgery |
| Diwakar A, et al. **[107]** | 11 | Male | Dyspnoea, palpitations | Not reported | Yes | Yes | No | Successful surgery |
| García-Saldivia M, et al. **[108]** | 32 | Female | Dyspnoea | LVH | Yes | Yes | No | Successful surgery |
| Sun J, et al. **[109]** | 11 | Male | Congestive heart failure | Not reported | Yes | Yes | BAV, aortic root aneurysm | Successful surgery |
| Li H, et al. **[110]** | 16 | Male | Dyspnoea | LVH | Yes | Yes | No | Successful surgery |
| Akbay Ş, et al. **[111]** | 1 day | Female | Congestive heart failure | Not reported | Yes | No | No | Successful surgery |
| Sui R, et al. **[112]** | 44 | Male | Dyspnoea. Chest tightness | First degree AV block, LVH | Yes | Yes | No | Successful surgery |
| Linnane N, et al. **[113]** | 8 months | Male | Heart murmur | Not reported | Yes | Yes | No | Successful interventional closure. Residual mild aortic regurgitation |
|  | 1 day | Male | Antenatal diagnosis | Not reported | Yes | Yes | Severe HOCM, LVNC, dysplastic aortic and pulmonary valves | Surgical repair with ultimate cardiac transplant for his remaining underlying cardiac anomalies |
|  | 10 months | Female | Heart murmur | Not reported | Yes | Yes | HLHS | Successful interventional closure + Status post Norwood/Sano and Glenn operations for HLHS |
|  | 9 months | Male | Heart murmur | Not reported | Yes | No | No | Successful interventional closure. Residual mild aortic regurgitation |
| Yang C, et al. **[114]** | 64 | Male | Dyspnoea | Atrial fibrillation. LVH. PVCs | Yes | Yes | No | Successful surgery |
| Mou HJ, et al. **[115]** | 6 | Female | Heart murmur | Not reported | Yes | Yes | No | Successful surgery |
| Wong AR et al **[116]** | 19 | Female | Heart murmur | Not reported | Yes | Yes | BAV | Left untreated |

**Acronyms:** DORV: double outlet right ventricle; ASD: atrial septal defect; PDA: patent ductus arteriosus; PS: pulmonary stenosis; BAV: bicuspid aortic valve; VSD: ventricular septal defect; RVOTO: right ventricular outflow tract obstruction; LVNC: left ventricular non compaction; HOCM: hypertrophic obstructive cardiomyopath2.

**Table 2.** PRISMA flow chart of selection process


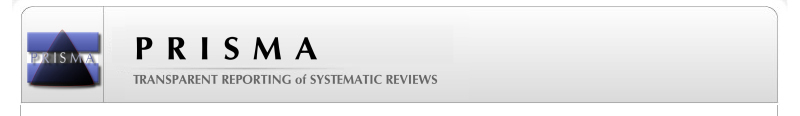
**PRISMA Flow Diagram**

## Identification

Records excluded
after checking the title

(n = 289)

(n = 4378)

Full-text articles excluded, after checking the abstract
(n = 70)

Duplicates removed
(n = 91)

## Included

## Eligibility

## Screening

Records screened
(n = 468)

Full-text articles assessed for eligibility
(n = 179)

Studies included in qualitative synthesis
(n = 109)

Studies included in quantitative synthesis
(n = 109)

Records identified through Pubmed and Scopus searching
(n = 559)
